# Randomised controlled trial of food elimination diet based on IgG antibodies for the prevention of migraine like headaches

**DOI:** 10.1186/1475-2891-10-85

**Published:** 2011-08-11

**Authors:** Natasha Mitchell, Catherine E Hewitt, Shalmini Jayakody, Muhammad Islam, Joy Adamson, Ian Watt, David J Torgerson

**Affiliations:** 1 York Trials Unit, Department of Health Sciences, University of York, York, YO10 5DD, UK; 2 Hull York Medical School, University of York, York, YO10 5DD, UK

**Keywords:** headache, diet, food elimination, randomised controlled trial

## Abstract

**Background:**

Research suggests that food intolerance may be a precipitating factor for migraine like headaches.

**Aim:**

To evaluate the effectiveness of the ELISA (Enzyme Linked Immuno-Sorbent Assay) Test and subsequent dietary elimination advice for the prevention of migraine like headaches.

**Design:**

Randomised controlled trial.

**Setting:**

Community based volunteers in the UK.

**Participants:**

Volunteers who met the inclusion criteria for migraine like headaches and had one or more food intolerance were included in the study. Participants received either a true diet (n = 84) or a sham diet (n = 83) sheet. Participants were advised to remove the intolerant foods from their diet for 12 weeks.

**Main outcome measures:**

Number of headache days over a 12 week period (item A MIDAS questionnaire). Other measures includes the total MIDAS score and total HIT-6 score.

**Results:**

The results indicated a small decrease in the number of migraine like headaches over 12 weeks, although this difference was not statistically significant (IRR 1.15 95% CI 0.94 to 1.41, p = 0.18). At the 4 week assessment, use of the ELISA test with subsequent diet elimination advice significantly reduced the number of migraine like headaches (IRR 1.23 95%CI 1.01 to 1.50, p = 0.04). The disability and impact on daily life of migraines were not significantly different between the true and sham diet groups.

**Conclusions:**

Use of the ELISA test with subsequent diet elimination advice did not reduce the disability or impact on daily life of migraine like headaches or the number of migraine like headaches at 12 weeks but it did significantly reduce the number of migraine like headaches at 4 weeks.

**Trial registration number:**

ISRCTN: ISRTCN89559672

## Introduction

Migraine is a condition associated with a severe one sided headache [1,2], which may be accompanied by nausea [3], vomiting, diarrhoea, blurry vision and photophobia [4]. Approximately 6-7% of men and up to 20% of women report experiencing migraine headaches [1]. It is considered by some that severe migraine can be as disabling as quadriplegia and is the cause of many General Practitioner (GP) consultations [5]. As well as having an impact on quality of life, migraine has a significant economic impact, with migraine sufferers requiring 4-6 bed rest days per year [6,7].

The aetiology of migraine attacks is not completely understood [8]. However, a number of precipitating factors have been identified in the literature including change in stress levels, excessive afferent stimuli, altered sleep patterns, weather change, and food [5,8].

The role of food in migraine has been a topic of scientific research since the early 1900s. Early studies found that elimination of specific foods from a person's diet could prevent the onset of a migraine or reduce the number of symptoms experienced [9,10]. More recent research suggests that food hypersensitivity (intolerance) may be a precipitating factor for migraine attacks [11] and about 25% of migraine patients report that their symptoms can be initiated by certain foods [12]. However, the role of food in migraine is still controversial. Unfortunately, the quality of the research (e.g. study design and sample size) generally in this field has not been very high [13-16]. Currently, the best accepted method for diagnosing and confirming food hypersensitivity is empirical, by elimination diet and challenge [17]. This method is laborious, and it is difficult to test all the combinations of food types that may be causing the problems. Previous studies which have looked at testing for food intolerance have focused on the presence of IgE antibodies, the "immediate response" [18,19]. An alternative approach would be to measure food specific IgG antibodies which characteristically exhibits a slower response [18,20]. The presence of food-specific IgGs may indicate a potential sensitivity to that particular food, previous studies have shown a relationship between IgG and food hypersensitivity [21-23]. Food specific antibody levels can be measured through the use of an Enzyme Linked Immuno-Sorbent Assay (ELISA), in the form of a simple blood test. The use of this test as the basis of food elimination diets is controversial with little evidence to support its use for migraines. A small cross over trial (n = 30) participants using ELISA testing has recently been reported, which demonstrated a significantly reduced frequency of headache days among a group of patients recruited from a headache clinic (27). In this study we undertook a further RCT of ELISA testing in a real life setting.

## Methods

The study was a single blind, two arm randomised controlled trial in which participants were randomised to either a "true" diet or "sham" diet control group. Participants were allocated to one of the two diet sheets based on a randomisation schedule developed using a random computer number generator by an independent data manager. Simple randomisation was used with no restrictions (e.g. blocking and/or stratification) placed on the randomisation. All participants and staff at YorkTest Laboratories were blinded to group assignment for the duration of the study.

### Identifying participants

Participants were recruited through advertisements and a press release. An advert for the trial was placed in the Migraine Action Association newsletter and on the University of York's webpage. Additionally there was a press release to the local media. Participants who expressed an interest in taking part in the study between March and June 2008 were sent further details about the study, a baseline questionnaire and a consent form. Because the participants were recruited through advertisement and through the completion of a postal questionnaire we could not be certain that all of them had a clinical diagnosis of migraine. Consequently, those recruited to the study did have self reported headaches that were 'migraine like'. Whilst most of the participants are likely to have had migraines it is possible that some did not. All participants aged 18-65, who had a self-reported diagnosis of migraine for at least 12 months, had no evidence of any other significant co-existing pathology and experienced 2 or more migraine like attacks (or 4 or more headache days) in the previous 4 week period were eligible for the screening phase. We wanted to recruit participants who were having regular migraines or migraine like headaches in order to maximise the power of the study.

At screening, potentially eligible participants were sent a pin prick test kit, supplied, at no cost, by YorkTest Laboratories Ltd (York, UK), with only a numerical identifier. Participants were asked to follow the instructions of the pin prick test in order to supply a small blood sample. Participants were asked to return this blood sample directly to YorkTest Laboratories. YorkTest Laboratories carried out an ELISA test to detect the presence of IgG antibodies specific to a panel of 113 different food antigens (see Table 1). Participants who had one or more food intolerances identified from the ELISA test were eligible for inclusion in the study. The cut-off used to determine food-specific IgG antibodies are detected or not was 10 AU (arbitrary units) per millilitre (AU/ml) of blood. Most of the 113 foods were tested individually. There were some that were tested as mixers (e.g., melon mix includes watermelon, honeydew and Cantaloupe melon). Participants were asked to remove all the foods in that particular mix (if appropriate). The test is CE marked and test reproducibility meets the requirements of the European IVD Directive. Participants who showed no evidence of food sensitivity in the ELISA test were excluded from randomisation and were informed of their true test results.

**Table 1:** Foodscan 113 - the foods tested

Dairy	Kiwi	Duck	Spinach
Cows milk	Lemon	Lamb	Lettuce

Egg white	Lime	Pork	Mustard mix

Egg yolk	Melon mix	Turkey	(cabbage, broccoli,

	(honeydew,		cauliflower,

**Fish**	watermelon,	**Nuts**	brussel sprout)

Crustacean mix	cantaloupe)	Almonds	Onion

(crab, lobster,	Olive	Brazil nut	Pea

prawn, shrimp)	Orange	Cashew	Pepper

Fish mix (cod,	Peach	Coconut	(capsicum)/paprika

haddock)	Pear	Hazelnut	Potato

Mollusc mix	Pineapple	Peanut	Soya bean

(oyster, mussel,	Plum	Walnut	String bean

scallop)	Raspberry		

Oily fish mix	Strawberry	**Grains**	**Other**

(mackerel, herring)	Tomato	Barley	Carob

Plaice/Sole		Buckwheat	Cocoa bean

Salmon/Trout	**Herbs/Spices**	Corn (maize)	Coffee

Tuna	Chilli pepper	Gluten (gliadin)*	Cola nut

	Cinnamon/clove	Millet	Hops

**Fruit**	Coriander/cumin/dill	Oat	Lentils

Apple	Garlic	Rice	Mushroom

Apricot	Mint mix (mint,	Rye	Sunflower seed

Avocado	sage, basil, thyme)	Wheat	Sesame seed

Banana	Mustard seed		Tea

Blackberry	Nutmeg/peppercorn	**Vegetables**	Yeast

Blackcurrant	Parsley	Asparagus	

Cherry	Vanilla	Aubergine	

Cranberry		Carrot	

Cucumber	**Meats**	Celery	

Grape	Beef	Haricot bean	

Grapefruit	Chicken	Kidney bean	

*A positive (IgG reaction) to gluten is not diagnostic of celiac disease

### Treatment and control group interventions

For each participant two diet sheets were generated by YorkTest Laboratories - a true diet sheet and a sham diet sheet. The sham diet sheet asked participants to eliminate the same number of foods to which they exhibited IgG antibodies but not those particular foods. The diet sheets were matched for the number of foods to be eliminated and also for the difficultly of eliminating foods. YorkTest Laboratories sent the true and sham diet sheets for each participant to the York Trials Unit, again with only a number for identification.

Participants were sent the appropriate diet sheet and were advised to remove foods to which they showed a positive reaction from their diet for a period of 12 weeks. At the end of this phase of the study, both groups were asked to reintroduce, in a stepwise fashion, the foods which they had been asked to eliminate. Participants were asked to reintroduce one food at a time on a weekly basis and continue with the food if no migraine or migraine like headache occurred. Food reintroduction occurred over a 4 week period with all eliminated foods (if no migraine or migraine like headache had occurred) being introduced in the final week. Participants were advised to stop consuming any reintroduced food if a migraine or migraine like headache occurred and to continue to reintroduce one of the other remaining foods. At the end of the follow up period all participants were told which diet sheet they had been given. Participants were sent the appropriate diet sheet to commence diet elimination in September 2008.

### Measurements

The baseline questionnaire recorded information about participants experience with migraine, including: frequency of migraines or migraine like headache in the previous 4 weeks (number of headache days), symptoms experienced, migraine medication use, whether they have previously excluded foods from their diet, and any consultations with healthcare professionals about their migraine or migraine like headache. The questionnaire also included a measure of disability using the MIgraine Disability Assessment Scale (MIDAS) [24] and impact on daily life using the Headache Impact Test (HIT-6™) [25,26]. Baseline questionnaires were sent out to participants at the beginning of July 2008. Reminders were sent 3 weeks later if participants had not returned their original questionnaire. Participants were followed-up at 4 weeks (October 2008) and 12 weeks (December 2008) after starting their allocated diet with a short questionnaire which included the MIDAS, HIT-6 and an additional question asking if the participant had consulted with a GP about their migraine or migraine like headache over the past 4 or 8 weeks depending on the timing.

The MIDAS questionnaire assesses headache-related disability. Respondents are asked to answer five questions, scoring the number of days, in the past 3 months, that they have had activity limitations due to headaches. Two additional items (A and B) are also included which ask about the number of headaches over the past 3 months and how painful the headaches were. The Headache Impact Test (HIT) is a tool to measure the impact headaches have on a person's ability to function on the job, at home, at school and in social situations. Respondents are asked to answer six questions covering aspects of functioning mostly impacted by headache: pain, role functioning (the ability to carry out usual activities), social functioning, energy or fatigue, cognition, and emotional distress. The responses on each question are described on a 5-point Likert scale including: never, rarely, sometimes, very often and always.

Participants in both groups completed a daily diary for the 12 weeks they were in the food elimination phase of the trial. The diary recorded whether or not they had a migraine or migraine like headache, the duration of the migraine or migraine like headache, the treatments they used and a migraine/headache severity score (0 to 10 where 0 is no pain and 10 is pain as bad as it can be). Participants in the "true" diet group were also asked to record their adherence to the diet each day in the diary. Participants were asked the following question: "Have you followed the study-related dietary advice today?". If participants answered "yes" to the question 70% of the time they were categorised as "adhering to the diet most of the time."

### Sample size

In a population survey of migraine sufferers it was found that on average patients had 13 migraines or headache days over a 12 week period (our follow-up period) with a standard deviation of 11 [24]. We sought a reduction of 5 headache days. Assuming a standard deviation of 11 this results in a standardised effect size (difference in means/standard deviation) of 0.45. This reduction of a 0.45 of a standard deviation was similar to that observed in a recent acupuncture trial for migraine prevention [2]. To detect this effect size, assuming 80% power, an independent samples t-test and a 2-sided 5% significance level we required 78 participants per group, 156 in total. Allowing for 10% loss to follow up we required a total of 174 participants (87 per group).

### Statistical analysis

The primary outcome measure was the number of headache days over a 12 week period (item A on the MIDAS questionnaire) which was calculated by summing responses on the 4 and 12 week questionnaires. A headache day was defined as any 24 hour period in which the patient reported that they had had a migraine attack which lasted more than 4 hours. Secondary outcome measures were the total MIDAS score and total HIT-6 score. The total MIDAS score was calculated by summing the total number of days from questions 1 to 5 (ignoring A and B). The HIT-6 scores were calculated by assigning a value of 6 to a response of "Never," 8 to "Rarely," 10 to "Sometimes," 11 to "Very Often," and 13 to "Always;" these values were then summed. Scores can range from 36 (lowest possible score) to 78 (highest possible score).

All analyses were conducted on an intention to treat bases, including all randomised patients in the groups to which they were randomised. Analyses were conducted in SAS (version 9.1) and SPSS (version 15). We compared the number of migraines in the intervention and control groups using negative binomial regression models with adjustments for baseline scores. These models are used to estimate the number of occurrences of an event when the event has Poisson variation with over-dispersion. We also used a negative binomial regression model to compare the total MIDAS scores in the intervention and control groups with similar adjustments for baseline scores. A linear regression model was used to compare the total HIT6 scores between the intervention and control groups after adjustment for baseline scores. Stricter levels of significance were used (p = 0.01) for secondary outcomes to compensate for multiple testing. Multiple imputation was used to account for the missing data. Five imputations were created using a set of appropriate imputation models constructed using variables that were predictive of the missing data (e.g. age, baseline number of headache days) and the group allocation. Multiple imputation was performed using the mi procedure in SAS with the assumption that the data were missing at random.

## Results

**Figure 1:**
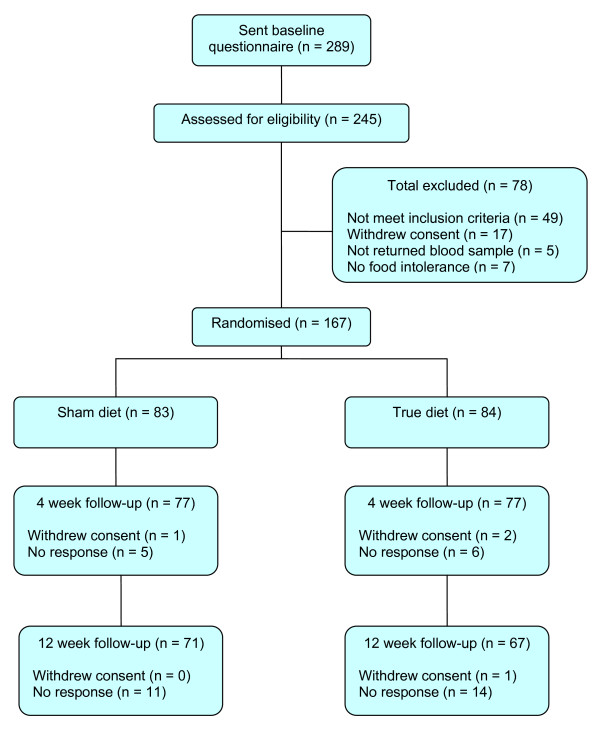
**Flow of participants**.

Recruitment of participants and their flow through the study is illustrated in Figure 1. In summary, between March 2008 and June 2008, 289 interested participants contacted York Trials Unit to register their interest in the study. Of the 289 questionnaires sent out, 245 (85% of interested participants) returned the questionnaire to be assessed for eligibility. Seventy eight participants were not randomised into the study as they did not meet the inclusion criteria, they withdrew consent, they did not return a blood sample for testing or they did not have any food intolerance identified by the ELISA test. A total of 167 participants (68% of eligible participants) were randomised, 83 to the sham diet and 84 to the true diet. At 12 weeks follow-up, 71 of the 83 participants in the sham diet group and 67 of the 84 participants in the true diet group returned their questionnaires (83% response rate).

### Participant characteristics

Table 2 shows the baseline demographic, clinical symptoms and contact with healthcare professionals of the two diet groups. The ELISA tested IgG antibodies specific to a panel of 113 different food antigens (see Table 1). These different foods were broadly categorized into 9 food groups: grains (e.g. wheat, oat, rye), dairy (e.g. cows milk, egg white), meats (e.g. pork, lamb), fish (e.g. salmon/trout, tuna), vegetables (e.g. lentils, soya bean, carrot), fruits (e.g. blackcurrant, peach, pineapple), nuts (e.g. cashew, brazil, peanut), herbs/spices (e.g. coriander/cumin/dill, chilli pepper, garlic) and others (e.g. cola nut, sunflower seed, tea). The frequency of food groups excluded from the diet is shown in Table 3. As Table 3 shows, generally, there was balance in the types of food eliminated across the two groups.

**Table 2:** Participant characteristics, reported symptoms and consulting health professionals

Characteristic	Sham diet (n = 83)	True diet (n = 84)	Total (n = 167)
Gender (females)	72 (87%)	75 (89%)	147 (88%)
Age (years), mean (sd)	47.1 (10.1)	48.3 (11.0)	47.7 (10.6)
No. migraines past 4 weeks, median (IQR)	5.0 (4.0 to 8.0)	6.0 (3.0 to 10.0)	6.0 (3.0 to 9.0)
Regular medication use	65 (79%)	67 (80%)	132(80%)

Symptoms experienced			
Intense throbbing headache	80 (96%)	80 (95%)	160 (96%)
Increased sensitivity to light	70 (84%)	72 (86%)	142 (85%)
Nausea	63 (76%)	69 (82%)	132 (79%)
Increased sensitivity to sounds	55 (66%)	53 (63%)	108 (65%)
Increased sensitivity to smells	42 (51%)	45 (54%)	87 (52%)
Visual disturbance	44 (53%)	43 (51%)	87 (52%)
Diarrhoea	21 (25%)	17 (20%)	38 (23%)
Other	34 (41%)	43 (51%)	77 (46%)

Consulted healthcare professional ^a^	83 (100%)	84(100%)	167(100%)
GP	82 (99%)	84 (100%)	166 (99%)
Hospital specialists	46 (55%)	42 (50%)	88 (53%)
Headache clinic	28 (34%)	26 (31%)	54 (32%)
CAM	49 (59%)	41(49%)	90 (54%)
Other	45 (54%)	32 (38%)	77 (46%)

Consulted GP in last 3 months			
Baseline (N = 167)	32 (39%)	37 (44%)	69 (41%)
3 months (N = 139)	20 (28%)	15 (22%)	35 (25%)

^a ^This refers to any consultation

**Table 3:** Frequency of foods

Food Groups	Sham diet	True diet
Grains	34 (41%)	40 (48%)

Dairy	72 (87%)	71 (85%)

Meats	28 (34%)	20 (24%)

Fish	6 (7%)	16 (19%)

Vegetables	27 (33%)	27 (32%)

Fruits	16 (19%)	23 (27%)

Nuts	47 (57%)	46 (55%)

### Primary outcome

The primary outcome was the number of headache days reported over the 12 weeks of diet elimination and was available for 69 of the sham and 69 of the true diet group (Table 4). The median number of headache days in the sham diet group was 20 (IQR = 12 to 29) and in the true diet group was 19 (IQR = 8 to 28). There was no significant difference in the reduction of migraines in the true and sham diet groups at 12 weeks (IRR 1.15 95% CI 0.94 to 1.41, p = 0.18). Similar results were found when multiple imputation was used to account for the missing data (IRR 1.17 95% CI 0.96 to 1.42, p = 0.11). When the data were analysed for the number of headache days at 4 weeks there was a significant difference between the two groups (sham n = 78, median = 8, IQR = 5 to 12; true n = 78, median = 7, IQR = 4 to 10; IRR 1.23 95% CI 1.01 to 1.50, p = 0.04). The finding was consistent when multiple imputation was used to account for the missing data (IRR 1.24 95% CI 1.02 to 1.50, p = 0.03).

**Table 4:** Number of headache days

Time point	Summary	Sham	True	IRR (95% CI)*	P-value
12 weeks**	N Median (IQR)	69 20 (12 to 29)	69 19 (8 to 28)	1.15 (0.94 to 1.41) *1.17 (0.96 to 1.42)*	0.18 *0.11*

4 weeks	N Median (IQR)	78 8 (5 to 12)	78 7 (4 to 10)	1.23 (1.01 to 1.50) *1.24 (1.02 to 1.50)*	0.04 0.03

*Values in italics are the results from using multiple imputation to account for the missing data

**Primary outcome

### Secondary outcomes

Secondary outcomes were the MIDAS and HIT-6, as can be seen from Table 5. Overall there was no significant difference between the true and sham diet groups in terms of disability as measured by the MIDAS or impact on daily life as measured by the HIT-6. The findings remained the same when multiple imputation was used to account for the missing data.

**Table 5:** Secondary outcomes

Outcome	Summary	Sham	True	IRR (95% CI)*	P-value
MIDAS	N Median (IQR)	61 14 (6 to 24)	54 12 (5 to 23)	1.22 (0.86 to 1.73) *1.16 (0.89 to 1.51)*	0.27 *0.26*

**Outcome**	**Summary**	**Sham**	**True**	Estimate (95% CI)*	**P-value**

HIT6	N, 4 weeks Mean (sd)	77 59.6 (7.8)	75 59.1 (9.0)	1.20 (-1.17 to 3.58) *1.25 (-1.11 to 3.60)*	0.32 *0.30*
	
	N, 12 weeks Mean (sd)	71 59.0 (7.0)	64 59.8 (6.7)	-0.11 (-2.24 to 2.02) *-0.28 (-2.17 to 1.60)*	0.92 *0.77*

*Values in italics are the results from using multiple imputation to account for the missing data

## Discussion

The use of the ELISA test to identify raised levels of IgG antibodies against tested foods with subsequent diet elimination advice reduced the number of migraines or migraine like headaches by 23% over 4 weeks. For our primary outcome measure, the results indicated a small decrease in the number of migraines or migraine like headaches over 12 weeks, although this difference was not statistically significant. We found little or no evidence that use of the ELISA test with subsequent diet elimination advice reduced the disability or impact on daily life as measured by the MIDAS and HIT-6 questionnaires.

### Strengths and weaknesses

We believe that this is the largest randomised controlled trial to investigate the use of diet elimination based on the presence of IgG antibodies for the prevention of migraine or migraine like headache. The use of ELISA testing has been investigated for IBS [18] and more recently for migraines [27]. One of the criticisms of the IBS trial was that the food eliminated from the sham diet did not appear to be as difficult as the true diet. In this study we tried to address this criticism and we feel that the comparability of the two diets was achieved. A potential weakness of the current trial, was that attrition was greater than we had originally anticipated (at 12 weeks n = 138, 17%). Another potential weakness of the study is that we recruited participants who self-reported their migraines. Hence, it is possible that they were not suffering from migraines but other forms of headache, which would not be amenable to dietary manipulation. Furthermore, some participants suffering from migraine or migraine like headache in our trial will not have been amenable to dietary manipulation as they will have suffered other 'triggers' for their migraines or headaches. The previous trial of diet elimination based in IgG testing for migraine [27] found a difference of 2.73 days at 6 weeks whilst we found a difference of 1 day at 4 weeks. The smaller effect in our study may have been explained by the fact our trial was more pragmatic and we did not provide dietary support for participants and we may have included some in the study who did not have 'true' migraines. Furthermore, because of low adherence with some participants not keeping to the recommended diet this would dilute any treatment effect. Nevertheless, despite this our findings do support this earlier trial.

Although food intolerance has been identified in the literature as a precipitating factor for migraine, it is one of many factors and the onset of a migraine may be triggered by either the intolerant food(s) or the combination of precipitating factors. However, other factors such as menstrual status, body mass index or ethnicity will have been balanced across by our use of randomisation. Vaughan [8] highlights few migraine patients can become headache free by dietary manipulation alone, therefore other precipitating factors need to be identified in order for them to be managed effectively [28]. In addition, for patients to experience any benefit from removing intolerant foods from their diet they obviously need to adhere to the diet change. The issue of adherence is further complicated by how aware a patient is of the use of their excluded food(s) in processed foods or when dining out. For example, mayonnaise may contain egg yolks, mustard, cornflour/maize starch, all three ingredients are possible foods that a patient could be asked to eliminate from their diet. To adhere to the diet a patient would need to read all food labels carefully to identify if the food contains any of their intolerant foods or ask at each dining establishment they ate at.

Within the current study only 52% of the participants in the true diet group returned their daily food diary. Whilst adherence was high among this group we do not know what it was like among the non-responders. Consequently, we were unable to assess the impact of adherence with the diet within the present study. Future research in this area needs to ensure that participants adherence with the dietary intervention is measured using a different tool rather than collecting this information through daily food diaries. Participants had to 'self-test' which is how this test is used in a real life. We could not be completely sure that samples related to the participant. However, it would seem unlikely that many, if any, participants would send another person's sample back.

Within the current study, all of the participants were informed that they would be given their true test results at 12 weeks, which may have led some participants, with more challenging diets, to wait until they knew their 'true' results before persevering with their diet. On the other hand, our participants were all volunteers so may be more highly motivated than the 'average' headache patient.

## Conclusion

In conclusion, use of the ELISA test with subsequent diet elimination advice did not reduce the disability or impact on daily life of migraines or migraine like headache or the number of migraines or migraine like headache at 12 weeks but it did significantly reduce the number of migraines or migraine like headache at 4 weeks. It might be that the avoiding the use of a sham may improve compliance as participants will know that they are allocated to a true diet and respond better to dietary advice.

Future trials in this area need to build in additional nutritional support for participants to aid adherence and need to identify a suitable tool to measure adherence with the dietary intervention.

## Abbreviations

AU: arbitrary units; AU/ml: arbitrary units per millilitre of blood; CE: Conformité Européenne (European Conformity); CI: Confidence interval; ELISA: Enzyme Linked Immuno-Sorbent Assay; GP: General Practitioner; HIT: Headache Impact Test; IBS: Irritable bowel syndrome; IgE: Immunoglobulin E; IgG: Immunoglobulin G; IQR: inter quartile range; IRR: incident rate ratio; IVD: in-vitro diagnostic; MIDAS: MIgraine Disability Assesment Scale; p: probability; RCT: Randomised controlled trial; SAS: Statistical analysis software; SPSS: Statistical Package for the Social Sciences.

## Ethical approval

The study was approved by the Research Governance Committee in the Department of Health Sciences, University of York, York, UK. Written informed consent was given by all participants.

## Authors' contributions

NM designed the study, coordinated the study and drafted the manuscript. CEH performed the statistical analyses and helped to draft the manuscript. SJ performed the statistical analyses. MH, JA and IW helped to draft the manuscript. DJT conceived of the study and helped to draft the manuscript. All authors read and approved the final manuscript.

## Competing interests

The authors declare that they have no competing interests.
